# Mesenchymal stem cells and myoblast differentiation under HGF and IGF-1 stimulation for 3D skeletal muscle tissue engineering

**DOI:** 10.1186/s12860-017-0131-2

**Published:** 2017-02-28

**Authors:** R. Witt, A. Weigand, A. M. Boos, A. Cai, D. Dippold, A. R. Boccaccini, D. W. Schubert, M. Hardt, C. Lange, A. Arkudas, R. E. Horch, J. P. Beier

**Affiliations:** 1 Department of Plastic and Hand Surgery and Laboratory for Tissue Engineering and Regenerative Medicine, University Hospital of Erlangen, Friedrich-Alexander University of Erlangen-Nürnberg (FAU), Krankenhausstraße 12, 91054 Erlangen, Germany; 2 Institute of Biomaterials, Department of Materials Science and Engineering, University of Erlangen-Nürnberg (FAU), Cauerstraße 6, 91058 Erlangen, Germany; 3 Institute of Polymer Materials, Department of Materials Science and Engineering, University of Erlangen- Nürnberg (FAU), Martensstrasse 7, 91058 Erlangen, Germany; 4 Interdisciplinary Clinic for Stem Cell Transplantation, University Cancer Center Hamburg (UCCH), 20246 Hamburg, Germany

**Keywords:** IGF-1, HGF, Mesenchymal stem cells, Myogenic differentiation, PCL-collagen nanofibers, Skeletal muscle tissue engineering

## Abstract

**Background:**

Volumetric muscle loss caused by trauma or after tumour surgery exceeds the natural regeneration capacity of skeletal muscle. Hence, the future goal of tissue engineering (TE) is the replacement and repair of lost muscle tissue by newly generating skeletal muscle combining different cell sources, such as myoblasts and mesenchymal stem cells (MSCs), within a three-dimensional matrix. Latest research showed that seeding skeletal muscle cells on aligned constructs enhance the formation of myotubes as well as cell alignment and may provide a further step towards the clinical application of engineered skeletal muscle.

In this study the myogenic differentiation potential of MSCs upon co-cultivation with myoblasts and under stimulation with hepatocyte growth factor (HGF) and insulin-like growth factor-1 (IGF-1) was evaluated. We further analysed the behaviour of MSC-myoblast co-cultures in different 3D matrices.

**Results:**

Primary rat myoblasts and rat MSCs were mono- and co-cultivated for 2, 7 or 14 days. The effect of different concentrations of HGF and IGF-1 alone, as well as in combination, on myogenic differentiation was analysed using microscopy, multicolour flow cytometry and real-time PCR. Furthermore, the influence of different three-dimensional culture models, such as fibrin, fibrin-collagen-I gels and parallel aligned electrospun poly-ε-caprolacton collagen-I nanofibers, on myogenic differentiation was analysed. MSCs could be successfully differentiated into the myogenic lineage both in mono- and in co-cultures independent of HGF and IGF-1 stimulation by expressing desmin, myocyte enhancer factor 2, myosin heavy chain 2 and alpha-sarcomeric actinin. An increased expression of different myogenic key markers could be observed under HGF and IGF-1 stimulation. Even though, stimulation with HGF/IGF-1 does not seem essential for sufficient myogenic differentiation. Three-dimensional cultivation in fibrin-collagen-I gels induced higher levels of myogenic differentiation compared with two-dimensional experiments. Cultivation on poly-ε-caprolacton-collagen-I nanofibers induced parallel alignment of cells and positive expression of desmin.

**Conclusions:**

In this study, we were able to myogenically differentiate MSC upon mono- and co-cultivation with myoblasts. The addition of HGF/IGF-1 might not be essential for achieving successful myogenic differentiation. Furthermore, with the development of a biocompatible nanofiber scaffold we established the basis for further experiments aiming at the generation of functional muscle tissue.

**Electronic supplementary material:**

The online version of this article (doi:10.1186/s12860-017-0131-2) contains supplementary material, which is available to authorized users.

## Background

Approximately one-half of our body consists of skeletal muscle, which is responsible for executing every single action we undertake [1]. Skeletal muscle has the ability to regenerate in response to damage by activating satellite cells resting beneath the basal lamina of adult skeletal muscle [2, 3]. However, this specific regeneration capacity is limited to only small wounds, whereas volumetric muscle loss caused by trauma or surgery requires remarkable efforts, such as free autologous muscle flap transplantation, which always come along with inevitable morbidity at the donor site [4–6]. This is where skeletal muscle tissue engineering (TE) might be a future goal, trying to mimic the structure and function of skeletal muscle [4–6].

For successfully generating muscle tissue in vivo, not only easily expandable cells but also a suitable biocompatible matrix needs to be generated. Muscle satellite cells offer the best characteristics for muscle TE, being capable of self-renewal and regeneration upon a variety of stimuli [7, 8]. However, multiple passaging decreases their differentiation capacity making their clinical applicability as single cell source difficult [9, 10]. Mesenchymal stem cells (MSCs) from the bone marrow may represent a promising alternative cell source for muscle TE since they can easily be harvested, expanded widely without losing their differentiation ability and autologous transplantation for future clinical applications does not come along with any risk of rejection [11, 12]. It has been described that MSCs can be differentiated towards the myogenic lineage by expressing muscle specific markers, even though their myogenic potential is limited [13–15]. Myogenic differentiation of MSCs alone might not be sufficiently satisfying, but they still represent an attractive cell source for co-cultivation with myoblasts. The application of MSCs co-cultivated with myoblasts has previously been investigated and it was shown that MSCs are able to fuse with myoblasts and contribute to the muscle regeneration process [13]. Moreover, it has been demonstrated that the addition of human MSCs to skeletal myoblasts cell-sheet in the ischemic cardiomyopathy model intensifies the release of different cytokines such as HGF and VEGF [16]. MSCs are not only known to secrete several growth factors involved in the muscle regeneration process such as basic fibroblast growth factor (bFGF), hepatocyte growth factor (HGF) or insulin-like growth factor 1 (IGF-1), but they also stimulate myoblast migration, proliferation, differentiation and cell survival upon co-cultivation [13, 16, 17]. Previous studies showed that stimulation with different supplements such as bFGF and dexamethasone potentiates MSC and myoblast differentiation capacity [18]. However, the effects of HGF and IGF-1 regarding the myogenic differentiation of MSC and myoblast co-cultures still require further investigation. It is well known that HGF activates satellite cells binding to the c-met tyrosine kinase receptor and stimulating different downstream targets [19]. While HGF primarily induces the proliferation of satellite cells, IGF-1 both activates proliferation and differentiation through binding to the IGF-1 receptor (IGF-1.R) [20]. The majority of circulating IGF-1 is bound to specific IGF-binding proteins (IGFBPs), a family of secreted proteins binding IGF-1 with greater affinity than IGF-1.R [21, 22]. There are different isoforms of IGFBPs and their exact roles are not clarified yet: While IGFBP4 mostly inhibits IGF stimulation, IGFBP5 acts through and independently of IGF and can therefore even potentiate or inhibit myogenic differentiation, and IGFBP6 is mostly expressed in proliferating cells [20, 21, 23, 24].

As mentioned above, successful generation of skeletal muscle needs both a suitable cell source as well as a biocompatible matrix. For optimally mimicking the in vivo structure of skeletal muscle and creating an applicable system for TE, a three-dimensional (3D) construct is needed. Different matrices have been studied for muscle TE applications, e.g. Heher et al. developed aligned fibrin fibrils in a 3D scaffold by applying static mechanical strain, demonstrating aligned myotube formation of myogenic precursor cells [25]. Further, Choi et al. demonstrated that cultivation of human skeletal muscle cells on unidirectional electrospun poly-ε-caprolacton (PCL)-collagen nanofiber meshes enhances myotube formation as well as skeletal muscle cell organization [26]. In previous studies, comparing fibrin-collagen-I gels with electrospun collagen nanofibers, good proliferation as well as differentiation of myoblasts could be shown, with parallel oriented nanofibers representing the most promising matrix [27].

One aim of this study is to investigate the influence of different concentrations as well as the combination of HGF and IGF-1 on myogenesis using co-cultures of MSCs and myoblasts as well as MSC monocultures. The three major myogenic key differentiation markers analysed in this study are, amongst others, myocyte enhancer factor 2 (MEF2), myosin heavy chain 2 (MyHC2) and alpha-sarcomeric actinin (ACTN2). MEF2 is a transcription factor, which interacts with members of the MyoD family of basic helix–loop–helix (bHLH) proteins to activate the skeletal muscle differentiation program. It plays a central role in activating pathways responsible for cell division, differentiation and death [28]. MEF2 is upregulated especially when cells enter the differentiation pathway and required in response to injury for adult myogenesis [18, 29–31]. However MEF2 seems to play a crucial role in myogenesis, its effects on MSC myoblast co-cultures have not been investigated profoundly so far. ACTN2, a cytoskeletal protein, stabilises the muscle contractile apparatus and is essential for developing the sarcomere. Such is, MyHC2, which constitutes sarcomere thick filaments and functions as a molecular motor protein in skeletal muscle. Both factors are indispensable for the formation of differentiated skeletal muscle [18, 32–34]. Their expression is proof for generating skeletal muscle. Even though, their behaviour concerning myogenic differentiation in MSC and myoblast co-cultures is not sufficiently studied and therefore of high interest.

We further analysed the behaviour of MSC-myoblast co-cultures in 3D fibrin and fibrin-collagen-I gels, especially in light of myogenic differentiation. As a final step, parallel-aligned electrospun PCL-collagen-I nanofibers were developed and cultivated with MSC-myoblast co-cultures stimulated with HGF and IGF-1 for testing the applicability for future in vivo studies.

## Methods

### Myoblast cell culture

Satellite cells were isolated from hind limb muscles of male Lewis rats (Charles River, Wilmington, Massachusetts, USA) as described previously [18]. For cell culture, Ham’s F-10 medium (Gibco, Carlsbad, California, USA) containing 25% FCS (Biochrom GmbH, Berlin, Germany), 1.25% Penicillin/Streptomycin (Biochrom GmbH) and 2.5 ng/ml bFGF (Peprotech, Hamburg, Germany) was used. The medium was changed every second day. Myoblasts of passage 3 were used for all experiments. To verify the myogenic phenotype of isolated cells, staining with the highly muscle-specific MyoD nuclear protein (5.8.A, Abcam, Cambridge, UK) was performed (see Additional file 1) [35].

### MSC cell culture

Rat MSCs were isolated from the bone marrow of male Lewis 1WR2 rats as described previously [36]. MSCs were stably transduced with green fluorescent protein (GFP) for cell labelling, and GFP-positive clones were expanded as described before by Lange et al. [36, 37]. Phenotype was assessed by their ability to differentiate into chondrocytes, adipocytes and osteocytes [36, 37]. MSCs were cultured in growth medium (DMEM Ham’s F-12, 10% FCS, 1% L-Glutamin, 1% P/S; all from Biochrom GmbH) and were used at passage 11 and 12 for all experiments. Medium was changed every second day.

### Differentiation conditions

Basic differentiation medium (DMEM/Ham’s F-12 + 2% donor horse serum (DHS) + 1% L-Glutamin + 1% P/S (Biochrom GmbH) + 0.4 μg/ml dexamethasone (Sigma Aldrich, St. Louis, Missouri, USA) + 1 ng/ml bFGF (Peprotech)) was supplemented with different concentrations of HGF (R&D Systems, Minneapolis, Minnesota, USA; 10, 30, 60, 100 ng/ml) and IGF-1 (Peprotech; 5, 10, 30, 60 ng/ml) and the combination of 10 ng/ml HGF + 10 ng/ml IGF-1. Cells were differentiated in mono- and co-cultures of myoblasts and MSCs for 2, 7 and 14 d (d = day). For co-culture experiments, cells were seeded in a ratio of 1:1 in 12-well culture plates at a density of 6 × 10^4^ cells in expansion medium (DMEM Ham’s F-12, 10% FCS, 1% L-Glutamin, 1% P/S). After 24 h, medium was replaced by differentiation medium. Medium was changed every second day.

For each experiment, myoblasts from three different isolations were used.

### Multicolour flow cytometry

Multicolour flow cytometry was carried out on a FACSCalibur cytometer with cell Quest software and analysed with Flowjo software (Tree Star, Ashland, Oregon, USA).

Mono- and co-cultures of myoblasts and MSCs were seeded at a density of 1.5 × 10^5^ in a 25-cm^2^ flask (Greiner, Frickenhausen, Germany) and cultured with differentiation media containing HGF 10 ng/ml and IGF-1 10 ng/ml and stimulated for 2 d and 14 d. Cells were detached and blocked in 5% FCS for 15 min. The pellet was picked up in 100-μl Cytofix/Cytoperm solution (Cytofix/Cytoperm Fixation/Permeabilization Kit; BD Biosciences, San Jose, California, USA) and incubated for 20 min at 4 °C. Cells were washed with BD Perm/Wash buffer. The cell pellet was incubated for 30 min at 4 °C with primary antibodies solved in 100-μl BD Perm/Wash Buffer in a concentration of 1:50 (anti-alpha-sarcomeric actinin (EA-53, Abcam), anti-MEF2 (MEF2A, B-4, Santa Cruz Biotechnology, Dallas, Texas, USA), all mouse-anti-rat IgG1). As a secondary antibody, PE anti-mouse IgG1 (BD Biosciences) was used (1:50, for 30 min at 4 °C). For further flow cytometry analysis, cells were picked up in PBS (Biochrom GmbH) with 2% FCS and 0.1% NaN_3_. Controls included unstained cells for negative and L6-myoblasts (L6-Mb) cell line (American Type Culture Collection, ATCC, Manassas, Virginia, USA) for positive control. As the isotype control, PE-labelled anti-mouse IgG1 (BD Biosciences) was used. For MSC and myoblast co-cultures as well as myoblast monocultures, myoblasts of three different isolations were used. Experiments with MSC monocultures were performed once.

### Immunocytochemistry

Cells of each group were seeded at a density of 1.2 × 10^4^ cells in expansion medium. After 24 h, the medium was replaced by differentiation medium. After fixation with ice-cold methanol, slides were washed and incubated in blocking buffer consisting of PBS with 1.5% FCS and 0.25% TritonX (Carl Roth GmbH, Karlsruhe, Germany) for 1 h at room temperature. After washing with TBS-T buffer (100 mM Tris and 60 mM NaCl in distilled water, 1 ml Tween20 per 1 L; pH 7.6), slides were covered with primary antibodies (anti-desmin (AB-1 (D33), Thermo Fisher Scientific, Runcorn, Cheshire, UK), anti-alpha-sarcomeric actinin (EA-53, Abcam), anti-MEF2 (MEF2A, B-4, Santa Cruz Biotechnology), anti-myosin heavy chain 2 (MYSN02, MyHC2, Thermo Fisher Scientific)) and diluted 1:50 in blocking buffer for 1 h at room temperature. As secondary antibody, Alexa Fluor 594 goat-anti-mouse IgG1 (Invitrogen, Karlsruhe, Germany) was used at 1:200 for 30 min at room temperature. Probes were counterstained with DAPI 1:1000 (Diamidine-phenylindole-dihydrochloride, Applied Science/Roche, Indianapolis; Indiana, USA) for 5 min. Slides were subsequently analysed and digitally photographed with a fluorescence microscope (IX83, cellSens software, Olympus, Hamburg, Germany). L6-Mb served as the positive control. An isotype control was performed using mouse IgG1 (BD Biosciences).

### RNA isolation and quantitative PCR analysis

In each group the expression rate of *DES (desmin), MYOG (myogenin), MEF2D (myocyte enhancer factor 2D), MyHC2 (myosin heavy chain 2), ACTN2 (alpha actinin skeletal muscle 2), IGFBP4, 5, 6* was analysed. As housekeeping gene *RPL13a (ribosomal protein L13a)* was used. RNA of all probes was extracted using the RNeasy Mini Kit (Qiagen GmbH, Hilden, Germany) according to the manufacturer’s protocols. RNA was reverse-transcribed into cDNA using a QuantiTect Reverse Transcription Kit and a Sensiscript Reverse Transcription Kit (both from Qiagen GmbH). cDNA was amplified through quantitative real-time PCR using SsoAdvanced Universal SYBR Green PCR Supermix (Bio-Rad, Hercules, California, USA) and Light Cycler (Bio-Rad iCycler iQ5). Probes were analysed in triplicates and variations of more than 1.5 threshold cycles were dismissed. Data evaluation was performed using the 2^-ΔΔCt^ method. The primer sequences used are given in Table 1.

**Table 1:** Primer sequences

	Forward primer	Reverse primer
*DES*	ATACCGACACCAGATCCAGTCC	TCCCTCATCTGCCTCATCAAGG
*MYOG*	TGAGAGAGAAGGGAGGGAAC	ACAATACACAAAGCACTGGAA
*MEF2D*	TGCTGCTCTCACTGTCACTAC	TTCACGACTTGGGGACACTG
*MyHC2*	TGACTTCTGGCAAAATGCAG	CCAAAGCGAGAGGAGTTGTC
*ACTN2*	TCACTGAGGCCCCTTTGAAC	AGACAGCACCGCCTGAATAG
*IGFBP4*	CAGCGTGCTTGCTAACTTCC	GCTTAGAGAACCAGACCCGG
*IGFBP5*	CCCTGCACCTGAGATGAGAC	TCACAGTTGGGCAGGTACAC
*IGFBP6*	AAGGCCCAGTCCTGTTCAAG	TGAGGTCACAGTTTGGCACA
*RPL13a*	CTCATGAGGTCGGGTGGAAG	AGAGCTGCTTCTTCTTCCGG

### Cell culture in 3D fibrin and fibrin-collagen-I gels

Fibrinogen and thrombin (Tisseel VH, S/D kit, Baxter AG, Vienna, Austria) were dissolved according to the manufacturer’s instructions. Collagen (rat tail collagen type I, BD Biosciences) for the fibrin-collagen gels was equilibrated to pH 7 prior to use. A co-culture of 100.000 CM-DiI (Invitrogen) labelled rat myoblasts and GFP-transduced rat MSCs at a ratio of 1:1 was mixed with either a fibrinogen-medium solution or fibrinogen-collagen-medium solution. Cell suspensions were mixed 1:1 with thrombin (final concentration of 6 IU) in a 24-well plate. Each gel had a total volume of 700 μl with a fibrin concentration of either 2.5 or 5 mg/ml. In the fibrin-collagen gels the collagen concentration was 0.25 mg/ml. The gels were finally covered with 400 μl of differentiation medium containing 0.1 TIU/ml aprotinin. After 2 and 7 d, gels were frozen in liquid nitrogen and minced with mortar and electrical mixer (IKA Werke, Staufen, Germany). Gels were further homogenised with Trizol (Life Technologies, Carlsbad, California, USA) and chloroform, and RNA was purified as described previously. A differentiation medium with and without HGF/IGF-1 was used.

### Electrospinning of PCL-collagen-I nanofibers and cell seeding

PCL (Sigma Aldrich) was dissolved at a ratio of 2:1 with bovine collagen type 1 (Symatese, Chaponost, France) in ethanol (VWR, Darmstadt, Germany) 90% at a concentration of 10% w/v (distance needle tip counter electrode: 20 cm). Parallel nanofibers were electrospun on a counter electrode consisting of two parallel arranged beams (distance between the beams: 3 cm) on a standard electrospinning machine (Linari, Pisa, Italy). Afterwards, fibres were collected from the beams using glass plates (1 cm diameter). Nanofibers were electrospun with a voltage of 20 kV and a flow rate of 1 ml/h. Twelve hours before cell seeding, probes sterilised in 70% ethanol, washed with PBS afterwards and soaked in DMEM Ham’s F-12 for approximately 1 h at 37 °C. Scaffolds were seeded with 100 μl expansion medium containing 50,000 MSCs and myoblasts at a ratio of 1:1. After an incubation time of 3 h at 37 °C, wells were filled with 1 ml of expansion medium. After 24 h, scaffolds were transferred into new well plates and stimulated with basic differentiation medium containing HGF and IGF-1 for 7 d. To analyse cell morphology and orientation, scanning electron microscopy and phase contrast microscopy (Olympus, Hamburg, Germany) was used.

### Scanning electron microscopy

Microstructural analysis of the scaffolds was performed using an Auriga Fib-SEM (Zeiss, Oberkochen, Germany). For this, the fibres were placed on aluminium stubs of 8 mm diameter. The probes were then sputter-coated with gold for 1 min using an EMITECH-K550 sputter coater at an operating pressure of 7 × 10^2^ bar and a deposition current of 20 mA. The SEM images were taken at an acceleration voltage of 2 kV and a working distance of approximately 8 mm.

### Time-lapse microscopy

GFP-MSC and CM-DiI-myoblasts were seeded in a ratio of 1:1 in 12-well culture plates at a density of 6 × 10^4^ cells in expansion medium. After 6 h, the medium was replaced by basic differentiation medium containing dexamethasone and bFGF. Culture plate was placed in an Olympus cell vivo microscopy system (IX83/cellVivo, cellSens software, Olympus, Hamburg, Germany). Co-cultures were cultivated under 37 °C and 5% CO_2_ for approximately 5 d. Four different positions were determined using the Olympus cellSens software. A picture of each position was taken every 10 min.

### Statistical analysis

Data are expressed as a mean–standard deviation. Statistical analysis was performed using SPSS 21.0 for Windows (SPSS, Chicago, Illinois, USA).

Results were statistically interpreted by one-way analysis of variance (ANOVA) and Tukey HSD test as a post hoc test. Normal distribution was confirmed using the Shapiro Wilk test. In the case of no normal distribution, the nonparametric Kruskal-Wallis test and the Mann-Whitney *U*-test were used. For comparing samples over different time points, ANOVA for repeated measurements was used.

The level of statistical significance was set to *p* ≤ 0.05. A *p-*value ≤ 0.01 was considered to be highly significant.

## Results

### Effects of HGF on mRNA level of different myogenic markers in MSCs co-cultivated with primary myoblasts and in monocultures

**Fig. 1:**
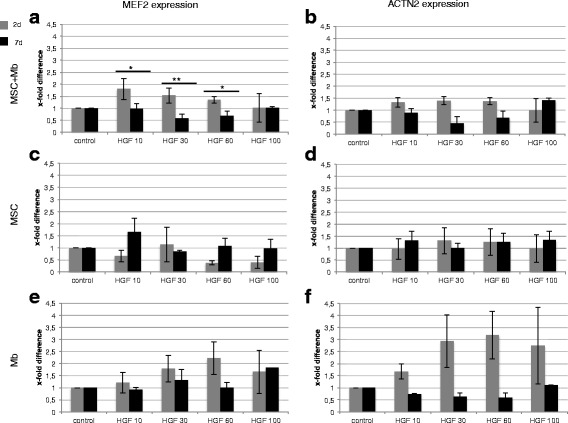
Expression of *MEF2* and *ACTN2* under different concentrations of HGF. Real-time PCR of MSC and myoblast (Mb) mono- and co-cultures under HGF stimulation as well as in unstimulated controls. Expressions are demonstrated in x-fold difference compared with unstimulated cells cultivated in basic differentiation medium (control = 1) using the 2^-ΔΔCt^ method. Markers are presented with mean +/- SD. **a** Significant and highly significant higher expression of *MEF2* in co-cultures after 2 d compared with 7 d using 10, 30 and 60 ng/ml HGF. **b** In co-cultures, *ACTN2* expression was upregulated during early stimulation compared with unstimulated control groups. 100 ng/ml HGF over 7 d induced the strongest *ACTN2* expression. **c** Strongest *MEF2* expression in MSC monocultures could be achieved with 10 ng/ml HGF after stimulation for over 7 d. **d** Seven-day stimulation with 10, 60 and 100 ng/ml HGF induced almost equal or higher levels of *ACTN2* in MSC monocultures compared with 2 d stimulation. **e**-**f** In Mb, a dose-dependent increase in *MEF2* (**e**) and *ACTN2* (**d**) expression was demonstrated from 10 to 60 ng/ml HGF during early stimulation. Increased levels of *MEF2* and *ACTN2* under HGF during early stimulation in co-cultures and Mb monocultures compared with unstimulated controls. Mb of three different isolations were used in three independent experiments. Three replicates of each were used. (** = p ≤ 0.01). (* = p ≤ 0.05)

MSC and primary rat myoblasts were co- and monocultured in basic differentiation medium containing HGF and in control medium without HGF for 2 d and 7 d. Expression of different myogenic markers could be observed under all conditions. In co-cultures, the early stimulation (2 d) with HGF demonstrated significant and highly significant upregulations of *MEF2* using 10, 30 and 60 ng/ml compared with late stimulation (7 d). A dose-dependent decrease of *MEF2* could be demonstrated after 2 d (Fig. 1a). Both *MEF2* and *ACTN2* expressions were equal or upregulated during early stimulation compared with unstimulated control groups (Fig. 1a–b). In MSC monocultures, the strongest *MEF2* expression (1.6 ± 0.6-fold) could be achieved with 10 ng/ml HGF after stimulation for over 7 d. Except in groups with 30 ng/ml HGF, long-term stimulation achieved almost equal or higher levels of *MEF2* and *ACTN2* in MSCs compared with controls (Fig. 1c–d). Varying results were observed in myoblast monocultures: Early stimulation with 10–60 ng/ml HGF induced a concentration-dependent upregulation of *MEF2* and *ACTN2* (Fig. 1e–f). Comparing the three different cell groups, it could be demonstrated that early stimulation with HGF increased the levels of myogenic markers especially in co-cultures and myoblast monocultures, whereas in MSCs this occurred during long-term stimulation.

### Effects of IGF-1 on mRNA level of different myogenic markers in MSC co-cultivated with primary myoblasts and in monocultures

**Fig. 2:**
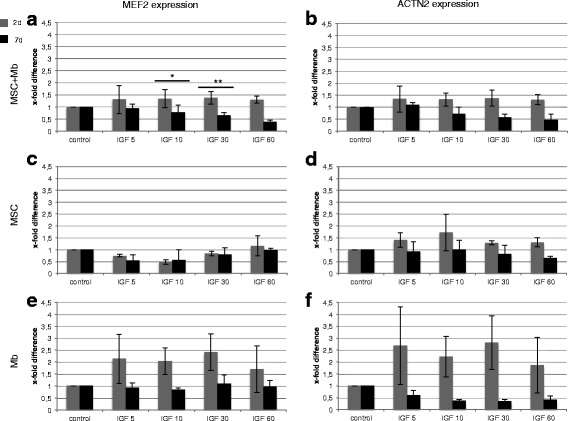
Expression of *MEF2* and *ACTN2* under different concentrations of IGF-1. Real-time PCR of MSC and myoblast (Mb) mono- and co-cultures under IGF-1 stimulation as well as in unstimulated controls. Expressions are demonstrated in x-fold difference compared with unstimulated cells cultivated in basic differentiation medium (control = 1) using the 2^-ΔΔCt^ method. Markers are presented with mean +/- SD. **a** Overall higher expressions of *MEF2* in co-cultures under the different IGF-1 concentrations compared with unstimulated conditions after 2 d. Significant and highly significant higher levels of *MEF2* after 2 d compared with 7 d using 10 and 30 ng/ml IGF-1. **b** Overall higher expressions of *ACTN2* in co-cultures under the different IGF-1 concentrations compared with unstimulated conditions after 2 d. **c** Stimulation with 60 ng/ml IGF-1 over 2 d induced the strongest upregulation of *MEF2* in MSC monocultures. **d** Overall increased *ACTN2* expression in MSC monocultures was observed during early stimulation, with highest levels under 10 ng/ml IGF-1. **e**-**f** Early stimulation with IGF-1 induced higher *MEF2* (**e**) and *ACTN2* (**f**) expressions in Mb monocultures compared with controls, with strongest expression under 30 ng/ml IGF-1. Increased MEF2 expression in co-cultures and Mb monocultures after 2 d compared with unstimulated controls. The highest *MEF2* and *ACTN2* levels were detected in Mb monocultures. Mb of three different isolations were used in three independent experiments. Three replicates of each were used. (** = p ≤ 0.01). (* = p ≤ 0.05)

MSC and primary rat myoblasts were co- and monocultured in basic differentiation medium containing IGF-1 and in control medium without IGF-1 for 2 d and 7 d. Expression of *MEF2* and *ACTN2* could be observed under all conditions. A highly significant and significant higher expression of *MEF2* could be detected in MSC and myoblast co-cultures after 2 d compared with 7 d in the 10 and 30 ng/ml IGF-1 group (Fig. 2a). *ACTN2* expression was upregulated during early stimulation compared with unstimulated control groups (Fig. 2b). In MSC monocultures, stimulation with 60 ng/ml IGF-1 over 2 d induced the strongest upregulation of *MEF2* (1.2 ± 0.4-fold) (Fig. 2c). *ACTN2* expression was overall increased during early stimulation (Fig. 2d). Mb monocultures were influenced positively by early stimulation with IGF-1: An overall increase of *MEF2* and *ACTN2* was observed after 2 d. Long-term stimulation showed no increase of myogenic markers (Fig. 2e–f). During early stimulation, *MEF2* expression increased in co-cultures and myoblast monocultures the most. Highest *ACTN2* expressions were seen in myoblast monocultures. The expression of *ACTN2* in MSC monocultures and co-cultures was similar.

### Influence of the combined stimulation of HGF and IGF-1 on mRNA level

**Fig. 3:**
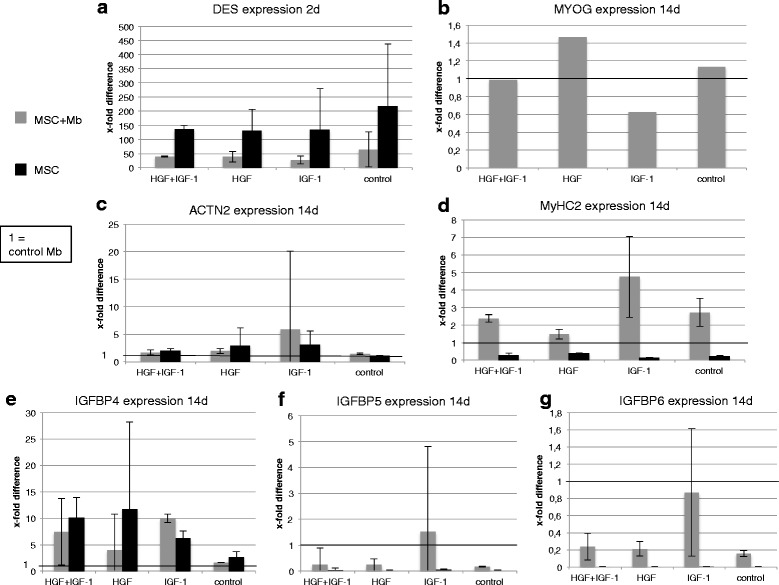
Expression of myogenic differentiation markers and IGFBPs under the influence of HGF and IGF-1. Real-time PCR of MSC and myoblast (Mb) mono- and co-cultures stimulated with HGF + IGF-1, HGF, IGF-1 or cultivated in unstimulated controls. Expressions are demonstrated in x-fold difference compared with Mb (=1) using the 2^-ΔΔCt^ method. Markers are presented with mean +/- SD. **a** After 2 d, the strongest *DES* upregulation was demonstrated in unstimulated controls. Throughout all conditions in MSCs, much higher levels of *DES* compared with co-cultures and Mb were observed. **b** After 14 d, strongest *MYOG* expression was detected under HGF stimulation compared with control myoblasts. *MYOG* could only be detected in one out of three experiments. **c** The highest expression of *ACTN2* was observed in IGF-1 stimulated groups, both in co-cultures and MSC monocultures. **d** The strongest upregulation of *MyHC2* in co-cultures was observed under IGF-1 stimulation. In MSC monocultures, the levels of *MyHC2* remained under all conditions lower than Mb. **e** In co-cultures, the highest *IGFBP4* levels were observed under IGF-1 stimulation. In MSC monocultures, HGF induced the strongest upregulation of *IGFBP4*. **f** In co-cultures, the highest *IGFBP5* levels were observed under IGF-1 stimulation. In MSC monocultures, the expression of *IGFBP5* remained lower under all conditions compared with Mb. **g** In both cell groups, the levels of *IGFBP6* were overall lower than in control Mb. Mb of three different isolations were used in three independent experiments. Three replicates of each were used

In MSC monocultures, as well as in co-cultures with myoblasts, higher expressions of *DES* compared with myoblasts (=1) could be observed. Cultivation in HGF/IGF-1 free medium achieved highest levels of *DES* (MSC: 218.5 ± 219-fold; MSC + Mb: 64.5 ± 62-fold) after 2 d. During early stimulation, MSC monocultures showed overall higher levels of *DES* than co-cultures (Fig. 3a). *MYOG* expression was upregulated under HGF stimulation compared with myoblasts under HGF after 14 d. *MYOG* could only be detected in one out of three experiments and merely in co-cultures (Fig. 3b). After 14 d, in co-cultures, highest levels of *ACTN2* (5.9 ± 14-fold) and *MyHC2* (4.8 ± 2.3-fold) could be observed under IGF-1 stimulation. *IGFBP4* expression increased in co-and monocultures with growth factors compared with cultivation in control medium without HGF/IGF-1 (Fig. 3e). *IGFBP5* and -*6* expression in co-cultures was elevated in the IGF-1 group compared with groups without growth factors. In MSC monocultures, hardly any expression of *IGFBP5* and -*6* could be detected (Figs. 3f–g).

### Myogenic differentiation and fusion of MSCs co-cultured with primary myoblasts under the influence of HGF and IGF-1

**Fig. 4:**
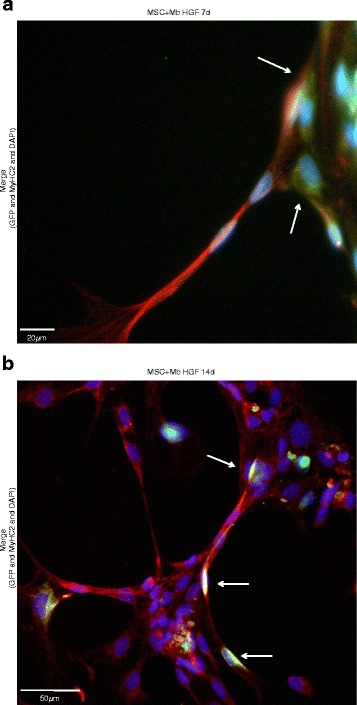
Fluorescence microscopy of MyHC2 in co-cultures. A positive staining of MyHC2, a muscle-specific major contractile protein, in MSC and Mb co-cultures under HGF stimulation for 7 d (**a**) and 14 d (**b**). Merge of DAPI (blue, nuclear staining), GFP (green, transduced MSC) and MyHC2 (red, with Alexa fluor 594 as secondary antibody). **a** The beginning formation of multinucleated cells could be observed under HGF stimulation. MSCs show positive expression of MyHC2 (arrows). Scale bars represent 20 μm. Magnification 400x. **b** The formation of multinucleated cells could be observed under HGF stimulation. It seems that MSCs are involved in the formation of multinucleated cells (arrows). Scale bars represent 50 μm. Magnification 200x

**Fig. 5:**
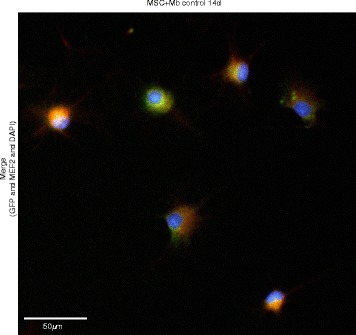
Fluorescence microscopy of MEF2 in co-cultures after 14 d. A positive staining of MEF2, a co-transcriptional factor expressed during muscle differentiation, in MSC and Mb co-cultures in control groups without HGF/IGF-1 after 14 d. Merge of DAPI (blue, nuclear staining), GFP (green, transduced MSC) and MEF2 (red, with Alexa fluor 594 as secondary antibody). The red fluorescence demonstrates the characteristic perinuclear localisation of MEF2. Scale bars represent 50 μm. Magnification 200x

With fluorescence microscopy, the myogenic differentiation potential of MSCs mono- and co-cultured with myoblasts was analysed under stimulatory and non-stimulatory effects after 7 and 14 d. A positive staining for muscle specific marker MyHC2, could be detected under HGF effects as well as the other tested conditions (Fig. 4a, b). MSC involvement in the formation of possibly multinucleated cells was verified by their green fluorescence protein expression, as these cells had been stable transduced prior to co-cultivation (Fig. 4b; arrows) (For single stainings see Additional files 2 and 3). Furthermore, myogenic differentiation could be demonstrated via positive staining for MEF2, expressed especially during muscle differentiation. Here, data of unstimulated controls are shown (Fig. 5) (For single stainings see Additional file 4).

**Fig. 6:**
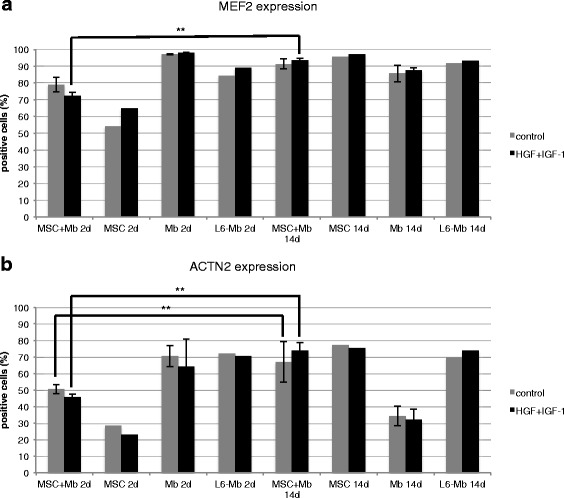
Flow cytometry analysis of MEF2 and ACTN2 in MSC and myoblast co-cultures, MSC and myoblast monocultures and L6-myoblasts. Markers are presented with mean +/- SD. **a** Highly significant upregulation of MEF2 in co-cultures from 2 to 14 d of stimulation with HGF + IGF-1. Higher levels of MEF2 in MSC monocultures could be observed in stimulated and control groups compared with L6-myoblasts. The expression of MEF2 was slightly downregulated after 14 d in myoblast (Mb) monocultures. **b** Highly significant upregulation of ACTN2 in co-cultures both under HGF + IGF-1 and in control groups after 14 d compared with 2 d. After 2 d of cultivation, the lowest levels of ACTN2 were demonstrated in MSC monocultures. A 2.7-fold upregulation in unstimulated controls and 3.2-fold under HGF + IGF-1 was observed in MSC monocultures after 14 d. The expression of ACTN2 was downregulated in stimulated and control Mb monocultures. Higher expression of ACTN2 was observed when Mb were cultivated in control groups. (** = *p* ≤ 0.01). Mb of three different isolations as well as three replicates of each were used. One replicate of MSC and L6 was used

Further myogenic differentiation was evaluated with flow cytometry analysis of MEF2 and ACTN2 (Fig. 6). MSC and myoblast co-cultures, MSC monocultures, myoblast monocultures and L6-Mb as positive controls were stimulated with HGF + IGF-1 or cultivated in control medium. After 14 d of cultivation, MEF2 expression in HGF + IGF-1 co-cultures showed a highly significant increase from 72.3% after 2 d up to 93.6% after 14 d. Expression of MEF2 in control groups did not increase significantly from 79% (2 d) to 91.3% (14 d). In MSC monocultures and L6-Mb, MEF2 expression was also upregulated after 14 d. Co-cultures and MSC monocultures achieved equal levels of MEF2 compared with L6-Mb after 14 d, both in HGF + IGF-1 and in control medium. Myoblast monocultures showed a decrease of MEF2 over time (Fig. 6a).

The expression of ACTN2 in co-cultures with HGF + IGF-1 was highly significantly upregulated (1.5-fold) from 2 to 14 d. Controls also showed a highly significant upregulation of ACTN2 from 50.6% after 2 d up to 67% after 14 d, but lower than the HGF + IGF-1 groups after 14 d (73.9%). In MSC monocultures, an increased ACTN2 expression was observed after 14 d compared with 2 d. Comparable to MEF2 expression, myoblasts showed a decrease in the myogenic marker over time. Co-cultures and MSC monocultures achieved equal levels of ACTN2 compared with L6-Mb after 14 d, both in HGF + IGF-1 and in the control medium (Fig. 6b).

Via microscope, we recorded the cell behaviour of MSC and myoblast co-cultures over a time period of 5 d. Signs of cell fusion between both cell sources could be seen (see Additional file 5). Considering that muscle repair and newly formation of skeletal muscle usually happens upon fusion of myoblasts, this might be a further step towards the generation of muscle tissue [17].



**Additional file 5:** Life-cell tracking of MSC and myoblast co-cultures. GFP-transduced MSCs (green) and CM-DiI-myoblasts (red) were co-cultivated in basic differentiation medium over a period of 5 d. Double-labelled cells indicate fusion of myoblasts and MSCs. (MPG 5416 kb)


### Effect of 3D scaffolds on myogenic differentiation of MSC and myoblast co-cultures

**Fig. 7:**
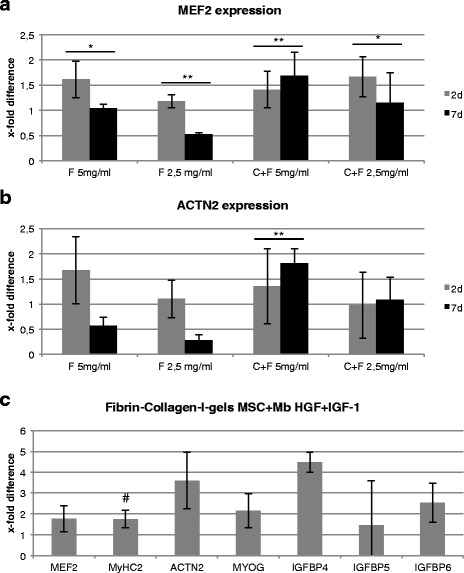
Myogenic differentiation in fibrin/fibrin-collagen-I gels. Markers are presented with mean +/- SD. **a** Real-time PCR of *MEF2* in MSC and myoblast (Mb) co-cultures cultivated in fibrin and fibrin-collagen-I gels. The expression of *MEF2* was highly significantly upregulated over time in 5-mg/ml fibrin-collagen-I gels. *MEF2* expression was significantly and highly significantly downregulated in other gel conditions. Expressions are shown in x-fold difference compared with co-cultures cultivated in 2D in control medium. **b** Real-time PCR of *ACTN2* in MSC and Mb co-cultures cultivated in fibrin and fibrin-collagen-I gels. The expression of *ACTN2* was highly significantly upregulated over time in 5-mg/ml fibrin-collagen I-gels. *ACTN2* expression was downregulated in other conditions, except 5-mg/ml fibrin-collagen-I gels with similar expression compared with the control. Expressions are shown in x-fold difference compared with co-cultures cultivated in 2D in control medium. **c** Real-time PCR of different myogenic markers (*DES, MEF2, MyHC2, ACTN2*) and *IGFBPs (IGFBP-4, -5, -6*) in co-cultures cultivated in fibrin-collagen-I gels and stimulated with HGF and IGF-1 for 2 d. Expressions are demonstrated in x-fold difference compared with unstimulated cells cultivated in control (=1). Upregulation of all myogenic markers under HGF + IGF-1 stimulation compared with unstimulated controls, MyHC2 significantly. (** = p ≤ 0.01). (* = p ≤ 0.05). (# = *p* ≤ 0.05 compared with unstimulated controls). Mb of three different isolations as well as three replicates of each were used

MSCs and myoblasts were cultivated in different 3D gels, consisting of either 5 or 2.5 mg/ml fibrin alone or in combination with collagen I. After 2 and 7 d, gene expression analysis of myogenic differentiation markers was performed. *MEF2* expression decreased significantly in 5 mg/ml fibrin gels and 2.5 mg/ml fibrin-collagen-I gels and highly significantly in 2.5 mg/ml fibrin gels over time, but not in 5 mg/ml fibrin-collagen-I gels, which experienced a highly significant increase (Fig. 7a).

Comparable to *MEF2, ACTN2* expression decreased over time in fibrin gels and increased highly significantly in 5 mg/ml fibrin-collagen-I gels (Fig. 7b).

A range of myogenic markers (*MEF2, MyHC2, ACTN2, MYOG, IGFBP4, -5, -6*) was analysed in co-cultures cultivated in HGF + IGF-1 for 2 d and compared with a differentiation medium without HGF/IGF-1. A slight, significant for *MyHC2*, upregulation of gene expression compared with control was detected (Fig. 7c).

**Fig. 8:**
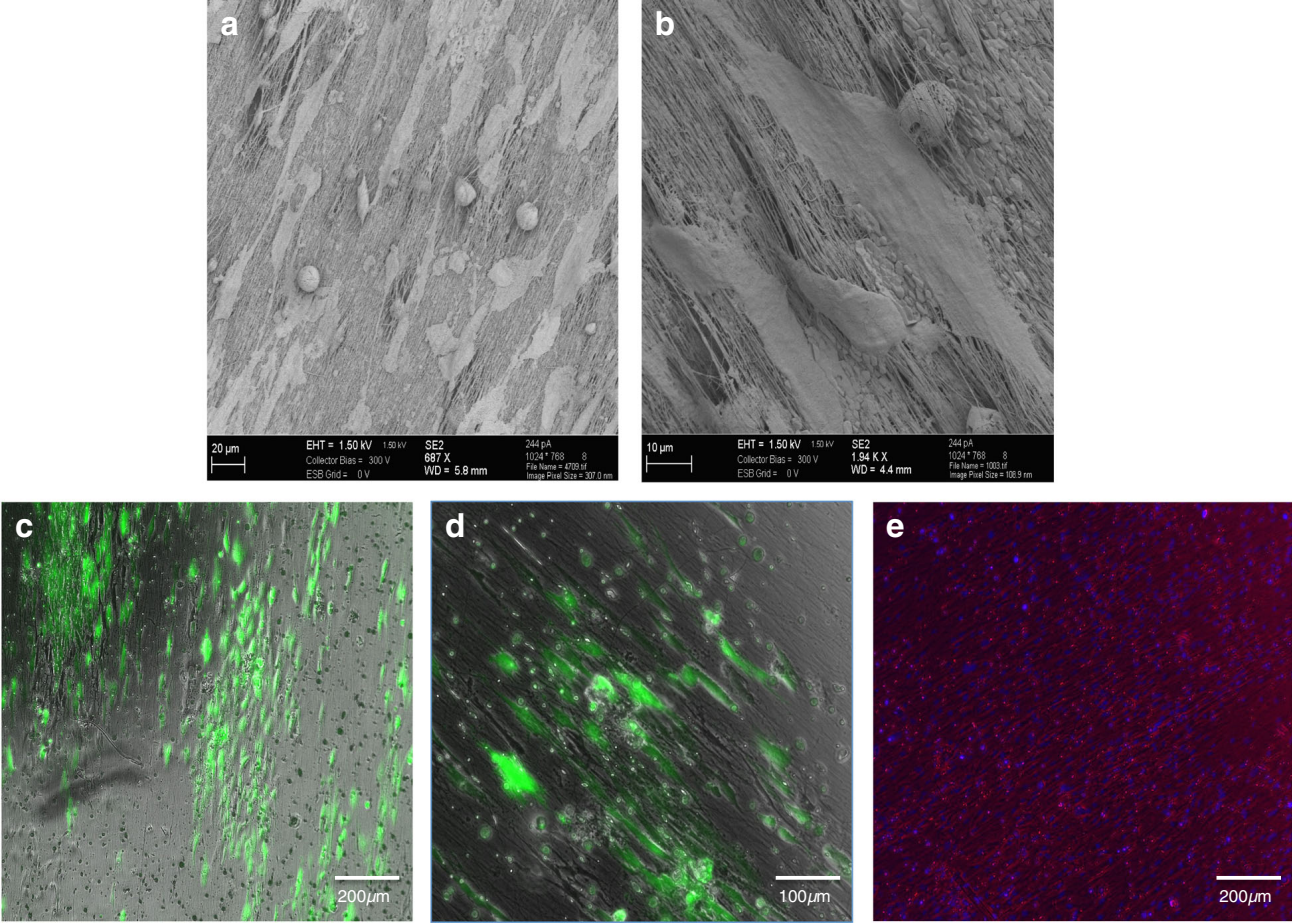
Cultivation of MSC-myoblast co-cultures on PCL-collagen-I nanofiber scaffolds. **a**-**b** SEM images of parallel-orientated PCL-collagen-I nanofiber scaffold cell attachments. **c**-**d** Parallel orientation of MSCs (green) on PCL-collagen-I nanofibers. **e** Positive staining for desmin (red) of MSC-myoblast co-cultures on PCL-collagen scaffolds. Nuclei were counterstained with DAPI (blue). Scale bars represent 200 and 100 μm. Magnifications 40x (**c**, **e**) and 100x (**d**)

MSC-myoblast co-cultures were further cultivated on parallel-aligned PCL-collagen-I-nanofiber scaffolds for 7 d and stimulated with HGF + IGF-1. SEM images of the PCL-collagen-I nanofibers showed a parallel-orientated scaffold (Fig. 8a, b). Using SEM and fluorescence microscopy, the attachment, proliferation and parallel alignment of the cells could be observed (Fig. 8c–d). Positive myogenic differentiation of cells growing on the scaffold could be demonstrated with desmin immunocytochemistry (Fig. 8e).

## Discussion

The aim of this study was to examine the effects of HGF and IGF-1 on MSC and myoblast co-cultures, as well as monocultures, and to investigate the cell–cell interactions in a 3D-matrix.

### Time-dependent effect of HGF and IGF-1 on myogenic differentiation of mono- and co-cultures

We investigated the influence of different concentrations of HGF and IGF-1 on MSC and myoblast co-cultures, as well as monocultures, compared with cells cultivated in HGF/IGF-1 free medium, analysing *MEF2* and *ACTN2* expression. Due to sometimes high standard deviations, some of the comparisons showed no statistically significant differences. An explanation for higher standard deviations might be that sometimes we could only isolate small amounts of RNA that perhaps do not fully reflect the total RNA of the analysed group and therefore caused variation among the experiments.

In our study, stimulation with different concentrations of the growth factors, revealed, especially under HGF, no clear trend regarding the expression of myogenic markers. It is well known that HGF both plays a role in proliferation as well as differentiation of skeletal muscle cells [38]. Yamada et al. described low expression of myogenin mRNA under 2.5 ng/ml as well as under extremely high (500 ng/ml) HGF concentrations, suggesting decreased myogenic differentiation [38, 39]. Walker et al. detected decreased Myosin Heavy Chain expression under 2 ng/ml HGF, but increased levels at 10 ng/ml, while Gal-Levi et al. showed lower MyHC levels with increased HGF concentrations (20–50 ng/ml) [38, 40]. A variety of studies have been made, trying to define the influence of different concentrations of HGF on skeletal muscle development, which has not been clarified yet.

In our study, early stimulation with HGF or IGF-1 achieved almost equal or increased levels of *MEF2* and *ACTN2* in co-cultures and myoblast monocultures, under all concentrations. Focussing on the temporal course, *MEF2* expression decreased significantly and highly significantly under HGF and IGF-1 influence in co-cultures. According to previously published findings, it may be suggested that too high concentrations of either of the added growth factors could negatively influence myogenic differentiation [41–44]. Differences were detected regarding MSC monocultures: especially long-term stimulation with HGF resulted in higher myogenic marker expression compared with unstimulated controls. During early stimulation in MSCs, only 30 ng/ml HGF achieved elevated levels of *MEF2* compared to unstimulated controls, being opposite to the other groups. Regarding that the result was not statistically significant, together with a high range of standard deviation and the fact that sometimes only small amounts of RNA could be isolated, these results might be of limited reliability. Furthermore, IGF-1 stimulation did not increase *MEF2* at all and *ACTN2* only during early time points. Comparing the level of myogenic differentiation amongst the three different cell groups, the co-cultures and MSC monocultures showed lower levels of myogenic marker expressions compared with myoblast monocultures, especially during early growth factor stimulation. The requirement of longer differentiation time periods for MSCs might be a possible explanation for lower myogenic marker expression during early time points. This is in accordance with previously published work, in which time periods up to 6 weeks were used [45, 46]. Expression of MEF2 and ACTN2 leads towards the formation of skeletal muscle [18, 29–33].

### Beginning myogenic differentiation of MSCs upon co-cultivation with myoblasts and under IGF-1 stimulation

We further analysed the effect of combined stimulation with HGF + IGF-1 compared with HGF or IGF-1 only, as well as under unstimulated conditions in a basal differentiation medium containing DHS, L-Glutamin, dexamethasone and bFGF (Fig. 3). Co-cultures and MSC monocultures were directly related to myoblast monocultures. During early stimulation, MSCs showed overall higher levels of *DES* compared with co-cultures, probably because of desmin being a MSC marker [47]. *MYOG* expression could only be detected in one out of three experiments and was limited to co-cultures, with highest levels under HGF stimulation. Long-term stimulation with IGF-1 showed increased levels of *ACTN2* and *MyHC2* in co-cultures, higher than in MSC and myoblast monocultures, indicating that MSCs might need longer differentiation periods [13, 48, 49]. Cell–cell contact between myoblasts and MSCs could be a possible explanation for increased myogenic marker expression in co-cultures, comparable to previous findings by Beier et al., in which elevated levels of MEF2 and ACTN2 were detected upon direct co-cultivation of MSCs and myoblasts [18]. In previous studies it has been described that MSC influence myoblast differentiation in a paracrine way [13, 16, 17].

With multicolour flow cytometry, the myogenic differentiation on a protein level was further analysed. Elevated levels of MEF2 and ACTN2 were detected in co-cultures and MSC monocultures after 14 d of stimulation with HGF + IGF-1 as well as in unstimulated controls indicating an increasing myogenic differentiation [30–33]. Under fluorescence microscopy, positive staining for MyHC2, part of the myosin motor protein and therefore responsible for skeletal muscle contraction, revealed further myogenic differentiation [50]. Through stable transduced GFP expression, it was possible to detect the involvement of MSCs in the formation of multinucleated cells (Fig. 4). Cultivation in HGF/IGF-1 free medium almost always achieved similar levels of myogenic differentiation than under HGF + IGF-1 stimulation. We were not able to detect significant differences between our stimulation and controls groups. Based on this observation, these growth factors may not necessarily be needed for sufficient myogenic differentiation [48, 49]. But then – what might be the explanation for adequate myogenic differentiation in HGF/IGF-1 free environment? First of all, our HGF/IGF-1 free medium (or control medium) contains already dexamethasone and bFGF, two factors known to influence myogenic differentiation [18, 51, 52]. Furthermore, as mentioned earlier, both MSC and myoblasts are known to secrete several growth factors involved in the muscle regeneration process [13, 16, 17, 43]. Herein, autocrine and paracrine stimulation might lead to myogenic differentiation. Nonetheless, successful muscle generation depends on more than secreted factors: cell-cell contact is crucial for a satisfactory differentiation. Previous works by Singaravelu and Padanilam compared the differentiation of MSC in conditioned medium with co-cultivation of MSC and injured renal cells. Cultivation in conditioned medium did not induce differentiation, but co-cultivation led to differentiation [53].

In summary, we successfully differentiated MSCs into the myogenic lineage both under HGF/IGF-1 stimulation and in a control medium, compared with myoblasts on the mRNA level (*MEF2, ACTN2, DES*) as well as after 14 d on the protein level (MEF2, ACTN2, MyHC2). Upon co-cultivation with myoblasts and under IGF-1 stimulation, additional expression of key myogenic marker *MyHC2* could be detected.

Comparing the myogenic potential of MSCs upon co-cultivation with myoblasts with MSC monocultures could be a promising future prospect. We already tried to separate MSCs by their GFP signal with fluorescence-activated cell sorting, but the yield was not enough for further analysis. In future experiments, it may be possible to sort MSCs for evaluation of myogenic potential cultivated in co- compared with monocultures, using higher cell numbers. Furthermore, only very low levels of *MYOG* could be detected in co-cultures in our study. MSC monocultures did not express *MYOG* at any time point. Because myogenin is mostly expressed during terminal stages of myogenic differentiation [54, 55], longer cultivation periods of at least up to 28 d would be one future goal. Although cell detachment after 28 d of cultivation made it impossible to analyse the gene expression during longer observation periods so far, coating with collagen type I or Maxgel™ (consisting of an undefined composition of human extracellular matrix components) may be a possibility to overcome cell detachment in future experiments [56]. Furthermore, myogenin is known to peak at some point of myoblast differentiation and then decline to lower expression afterwards. By the time we analysed myogenin expression, it might be possible that its expression was already starting to decline [57, 58].

### Possible involvement of IGFBPs in myogenic differentiation

IGFBPs are a family of secreted proteins binding IGF-1 and either potentiating or inhibiting IGF-1 actions on myogenic differentiation [20, 22]. In our study, in co-cultures, increased expression of *IGFBP4, - 5* and -*6* goes along with higher *ACTN2* and *MyHC2* expression under IGF-1 stimulation compared with myoblasts and MSC monocultures, accompanied by lower expressions of *DES* and *MYOG* (*MYOG* was only detected in one out of three experiments in co-cultures). *IGFBP5* and *-6* showed a similar expression pattern amongst all conditions in co-cultures, suggesting that these genes might have equal effects on myogenic differentiation and are regulated alike. Furthermore, the expression of *MyHC2* and *ACTN2* appears to correlate with *IGFBP5* and *-6* in co-cultures, indicating that they might have a positive influence on the expression of those myogenic markers.

In MSC monocultures both under HGF + IGF-1 and HGF stimulation, elevated levels of *IGFBP4* as well as *ACTN2* were observed compared with co-cultures and myoblast controls, whereas *IGFBP5, -6* and *MyHC2* expressions were almost undetectable.

Depending on the culture conditions (co-/monocultures), growth factor stimulation and the analysed myogenic markers, different effects could be detected. In co-cultures under IGF-1, increased expression of *IGFBPs* was observed together with elevated levels of *ACTN2* and *MyHC2*, and MSC monocultures showed different results under the same conditions. Hence, the function of the different IGFBPs might vary among different surrounding conditions. So far, we presume that IGFBPs play a role during myogenic differentiation.

Even though there is still no uniform opinion concerning the exact function of the IGFBPs, IGFBP4 was both identified as a positive influencer during muscle regeneration and as a potent inhibitor of muscle growth and IGF-1 actions [20, 21, 59, 60]. IGFBP5 could inhibit IGF-1 actions, potentiate IGF-1 effects or act in an IGF-independent way [24, 61]. IGFBP6 may not act primarily during the myogenic differentiation process [20].

Using ELISA or Western Blot, the concentrations of IGFBPs in the cell lysate or the supernatant could be estimated more precisely. Inhibiting IGFBPs through IGFBP antibodies could be another approach to gain more information about these binding proteins and their effects on IGF-1 and myogenic differentiation.

### Three-dimensional environment enhances myogenic differentiation of MSCs and myoblasts

Regarding the matrix evolution in TE, we previously demonstrated that 3D collagen-I gels had a stimulatory effect on myoblasts [27]. According to these findings, we investigated the effect of 3D systems on co-cultures of MSCs and myoblasts with HGF + IGF-1, and observed a strong upregulation of myogenic key markers compared with unstimulated groups. However, fibrin-collagen-I gels cannot provide the needed spatial orientation for muscle tissue. Therefore, we developed an electrospun, parallel-aligned, PCL-collagen-I nanofiber scaffold as the basis for further generation of muscle tissue. Parallel alignment of fibres stimulates myotube formation, and the combination of PCL and collagen provides strength, elasticity and compliance, which is essential for the formation of functional tissue [26, 62]. Cultivating MSCs and myoblasts on parallel-oriented PCL-collagen-I nanofibers and stimulating with HGF + IGF-1 for 7 d leads to parallel alignment of the cells in this study, indicating that this scaffold is a promising matrix for generation of muscle tissue in vitro. Jana et al. cultivated C2C12 myoblasts on aligned chitosan-PCL hybrid nanofiber scaffolds, showing formation of a compact assembly of myotube cells [63]. Zhao et al. used aligned electrospun PCL/collagen hybrid scaffolds for diaphragmatic repair in rats, demonstrating muscle cell migration and tissue formation [64]. For further investigation of our results in vivo, the newly developed arteriovenous loop model combined with nervous innervation through the saphenous nerve might offer a promising possibility for the functionalization of skeletal muscle [65].

Although promising results for engineering of vascularised tissue have already been achieved in the case of bone reconstruction [66], free autologous muscle flap transplantation still remains the gold standard for muscle reconstruction, in particular for complex soft tissue defects [67]. However, in the future, TE of skeletal muscle may help to overcome the donor site problem of harvesting large muscles of the human body.

## Conclusions

The generation of functional skeletal muscle tissue for future in vivo applications is still challenging. In this study we demonstrated that MSCs in monocultures and in co-cultivation with myoblasts are able to differentiate into the myogenic lineage by expressing key myogenic markers such as desmin, MEF2, MyHC2 and ACTN2. Stimulation with HGF and IGF-1 induces an upregulation of different myogenic markers, but probably is not essential for myogenic differentiation. IGFBPs play a role during myogenic differentiation, varying amongst culture and stimulation conditions. Three-dimensional cultivation of co-cultures enhances the myogenic differentiation capacity. PCL-collagen nanofibers especially represent a promising scaffold, mimicking the structure of skeletal muscle and inducing parallel alignment of MSCs and myoblasts. The results of this study represent important starting points for future studies and in vivo applications for the TE of skeletal muscle.

## Additional files

Additional file 1: Fluorescence microscopy of MyoD. Primary rat myoblasts after being passaged two times since isolation. A merge of DAPI (blue) and MyoD (red, with Alexa fluor 594 as secondary antibody) staining is shown. (PDF 472 kb)
Additional file 2: Fluorescence microscopy of MyHC2 after 7d. Single stainings of MyHC2 in MSC and Mb co-cultures under HGF stimulation for 7 d. (a) Nuclear staining with DAPI. (b) GFP-transduced MSC in green colour. (c) Staining for MyHC2 with Alexa fluor 594 as secondary antibody. Scale bars represent 20 μm. Magnification 400x. (PDF 2443 kb)
Additional file 3: Fluorescence microscopy of MyHC2 after 14d. Single stainings of MyHC2 in MSC and Mb co-cultures under HGF stimulation for 14 d. (a) Nuclear staining with DAPI. (b) GFP-transduced MSC in green colour. (c) Staining for MyHC2 with Alexa fluor 594 as secondary antibody. Scale bars represent 50 μm. Magnification 200x. (PDF 1334 kb)
Additional file 4: Fluorescence microscopy of MEF2. Single stainings of MEF2 in MSC and Mb co-cultures in control groups without HGF/IGF-1 after 14 d. (a) Nuclear staining with DAPI. (b) GFP-transduced MSC are in green colour. (c) Staining for MEF2 with Alexa fluor 594 as secondary antibody. Scale bars represent 50 μm. Magnification 200x. (PDF 792 kb)

## Abbreviations

2D: Two-dimensional
3D: Three-dimensional
ACTN2: Alpha-sarcomeric actinin
bFGF: Basic fibroblast growth factor
d: Days
DAPI: Diamidine-phenylindole-dihydrochloride
DES: Desmin
DHS: Donor horse serum
FACS: Fluorescence-activated cell sorting
GFP: Green Fluorescent Protein
h: Hours
HGF: Hepatocyte growth factor
IGF-1: Insulin-like growth factor-1
IGF-1.R: IGF-1 receptor
IGFBPs: IGF-binding proteins
Mb: Myoblasts
MEF2: Myogenic Enhancer Factor 2
MSC: Mesenchymal stem cells
MyHC2: Myosin heavy chain
MYOG: Myogenin
PCL: Poly-ε-caprolacton
RPL13a: Ribosomal protein L13a
RT-PCR: Real-time PCR
SEM: Scanning Electron Microscopy
TE: Tissue engineering
