# Supplementary figures and images for: Mesenchymal stem cells and myoblast differentiation under HGF and IGF-1 stimulation for 3D skeletal muscle tissue engineering
Source: BMC Cell Biol. 2017 Feb 28;18:15. doi: 10.1186/s12860-017-0131-2 (PMC5331627; doi:10.1186/s12860-017-0131-2)

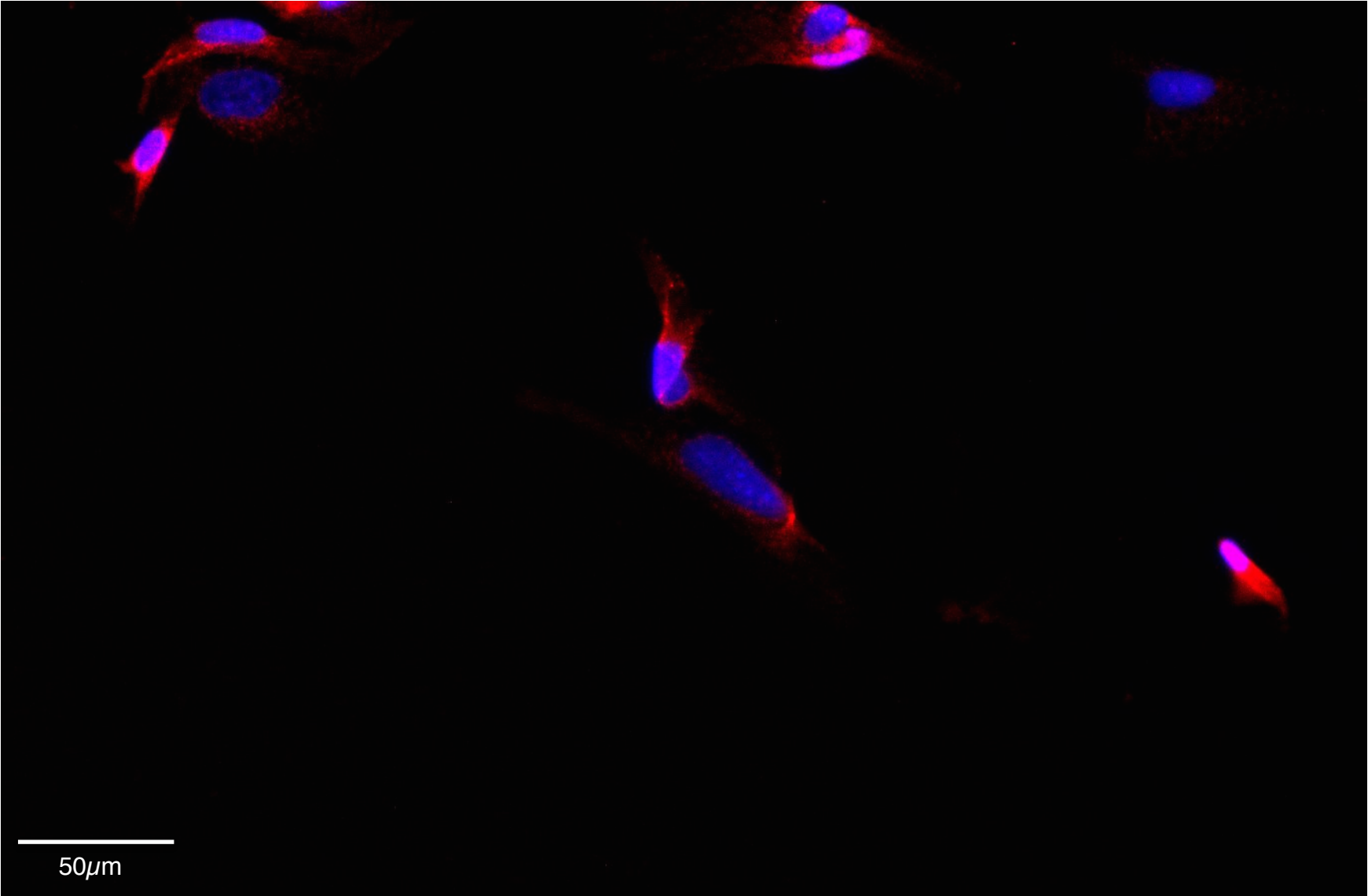

Supplement: Additional file 1: — Fluorescence microscopy of MyoD. Primary rat myoblasts after being passaged two times since isolation. A merge of DAPI (blue) and MyoD (red, with Alexa fluor 594 as secondary antibody) staining is shown. (PDF 472 kb) [file 12860_2017_131_MOESM1_ESM.pdf]

DAPI

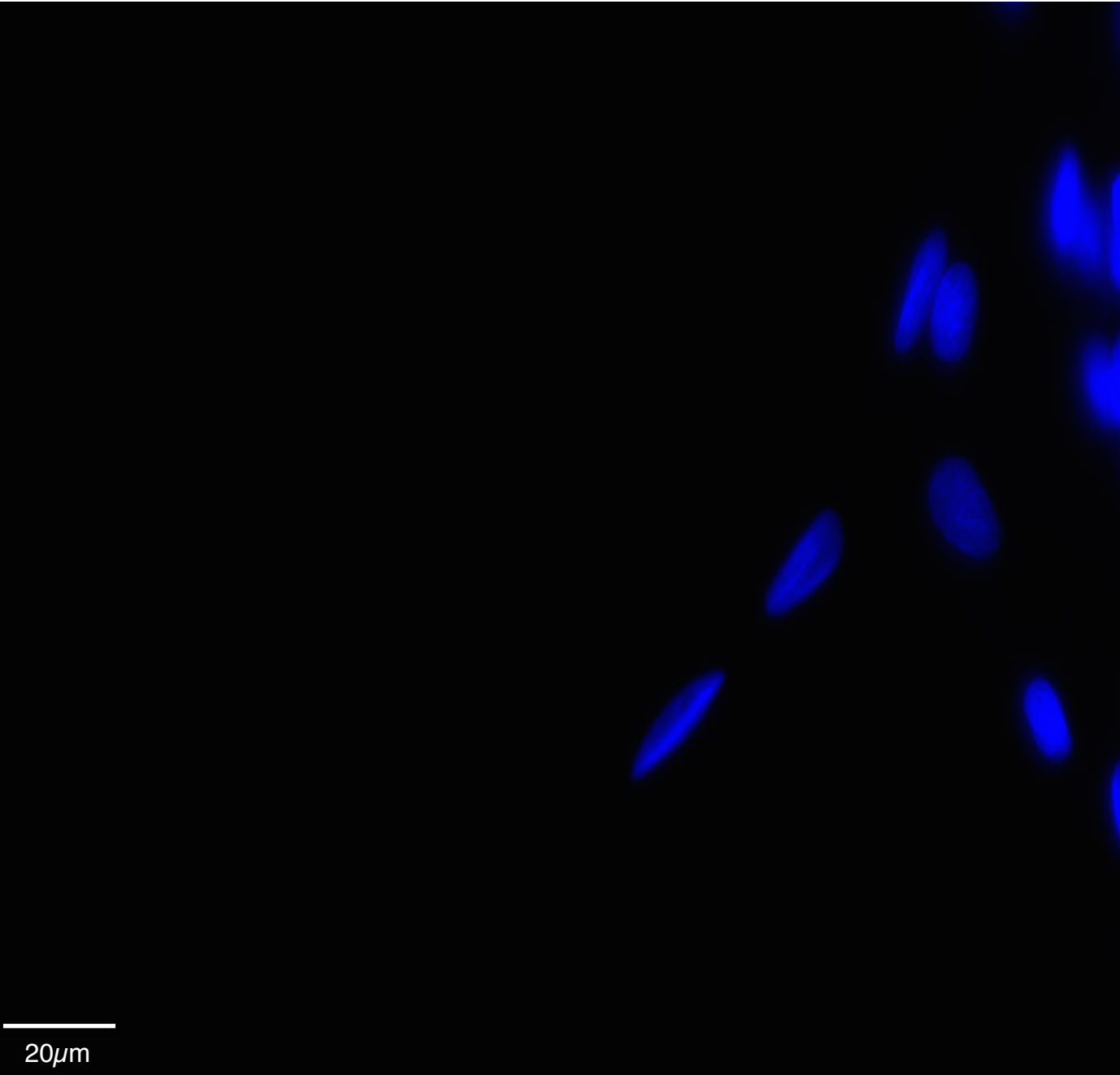

20μm

GFP

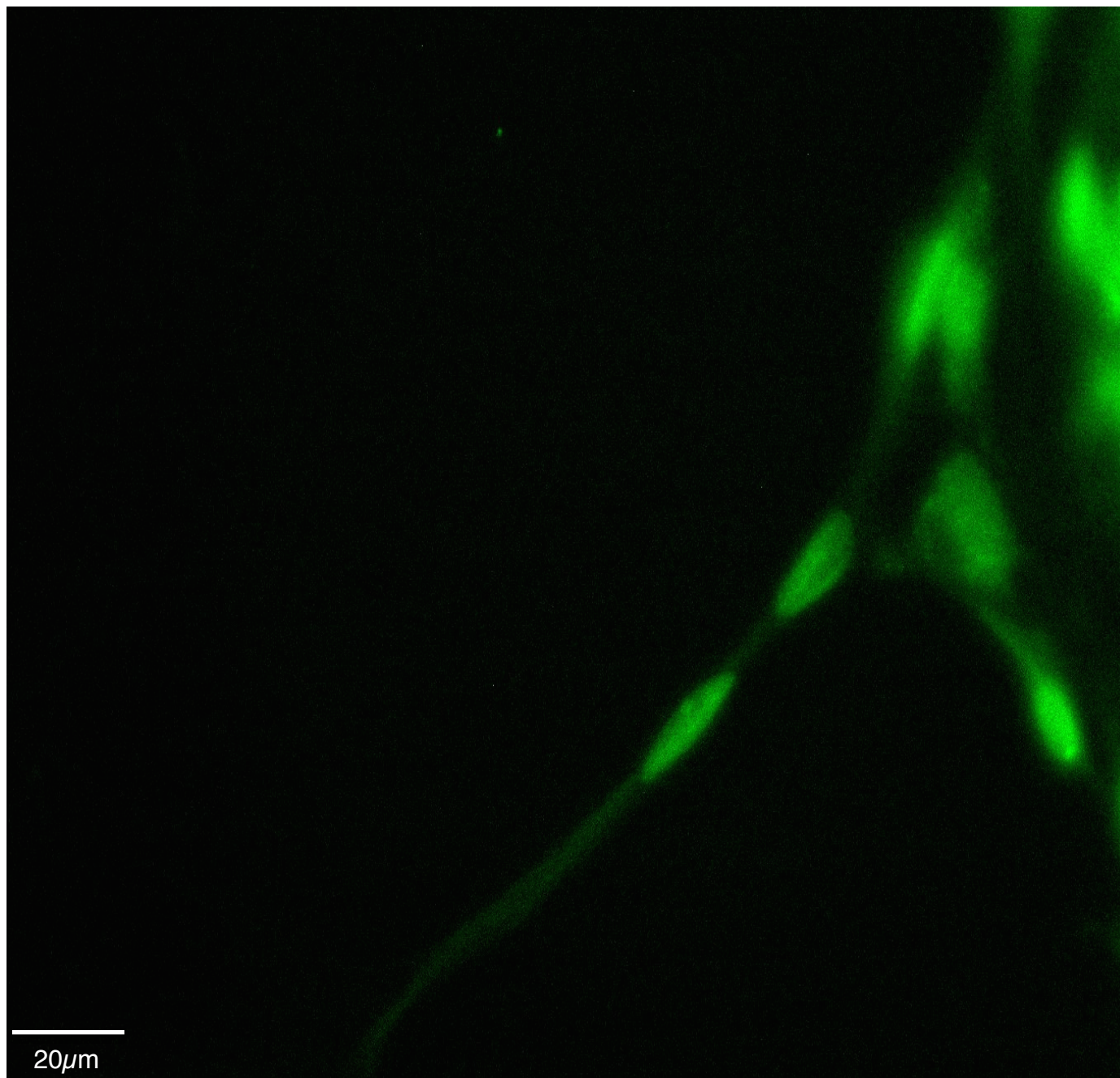

MyHC2

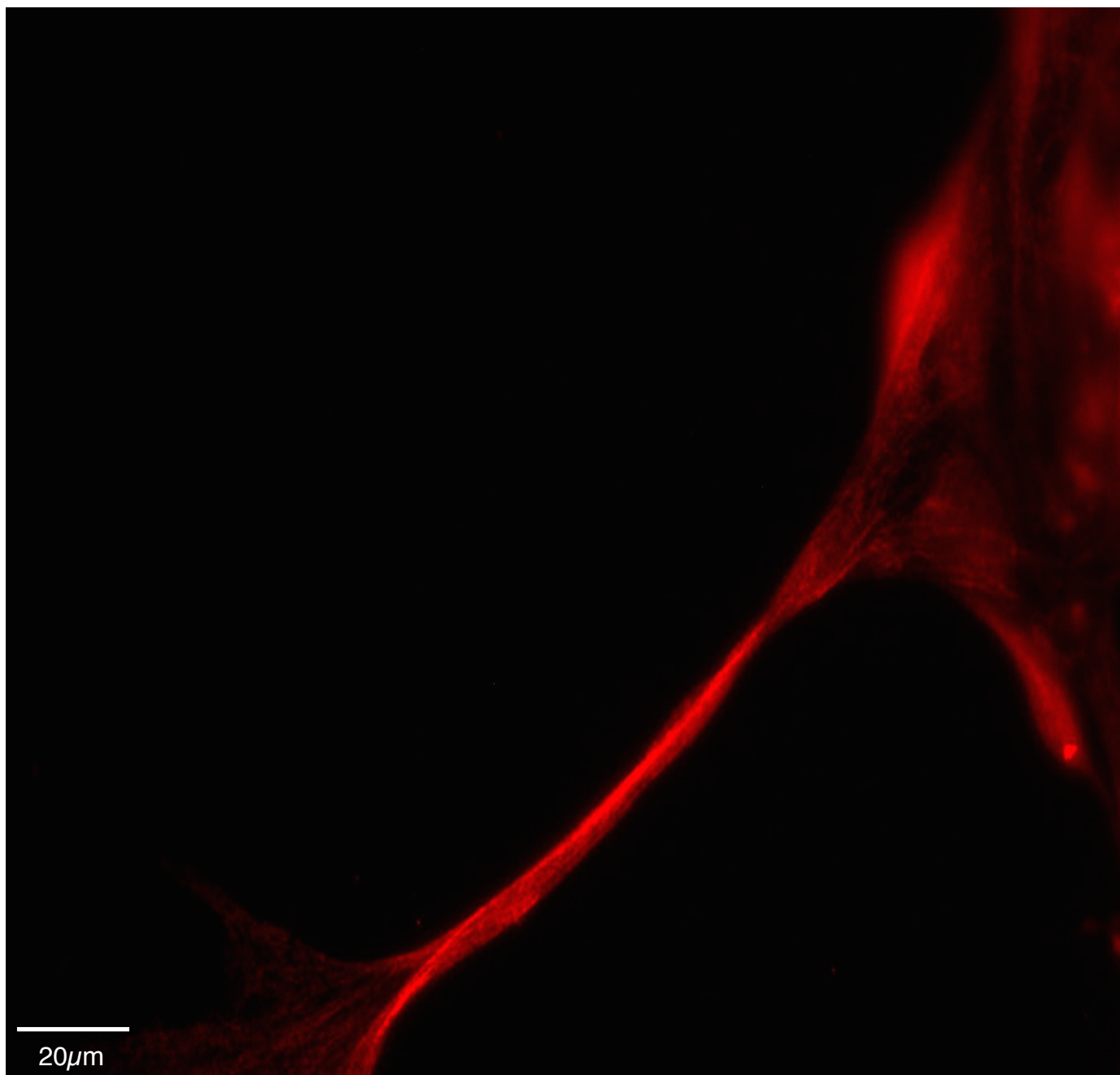

Supplement: Additional file 2: — Fluorescence microscopy of MyHC2 after 7d. Single stainings of MyHC2 in MSC and Mb co-cultures under HGF stimulation for 7 d. (a) Nuclear staining with DAPI. (b) GFP-transduced MSC in green colour. (c) Staining for MyHC2 with Alexa fluor 594 as secondary antibody. Scale bars represent 20 μm. Magnification 400x. (PDF 2443 kb) [file 12860_2017_131_MOESM2_ESM.pdf]

DAPI

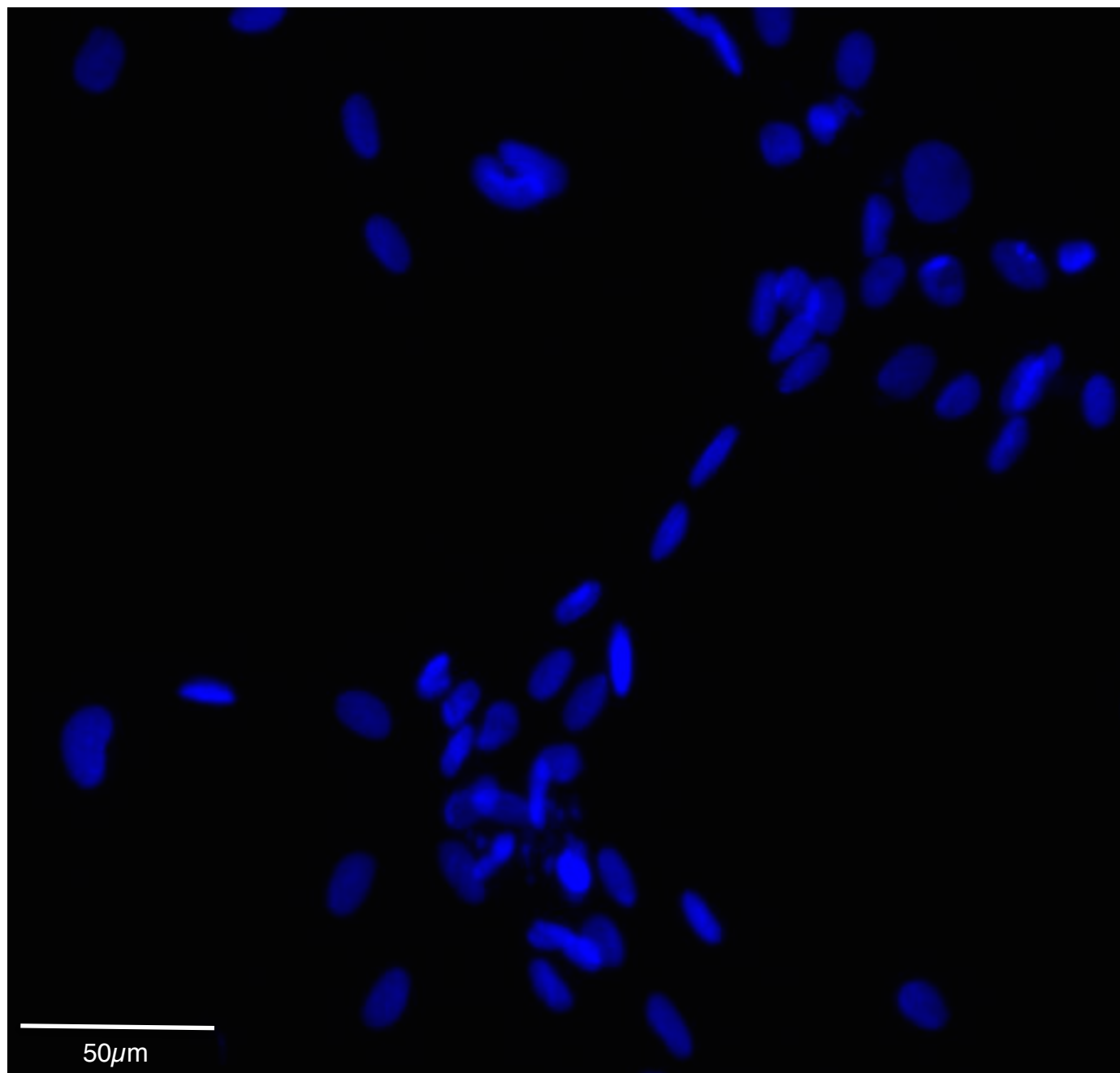

GFP

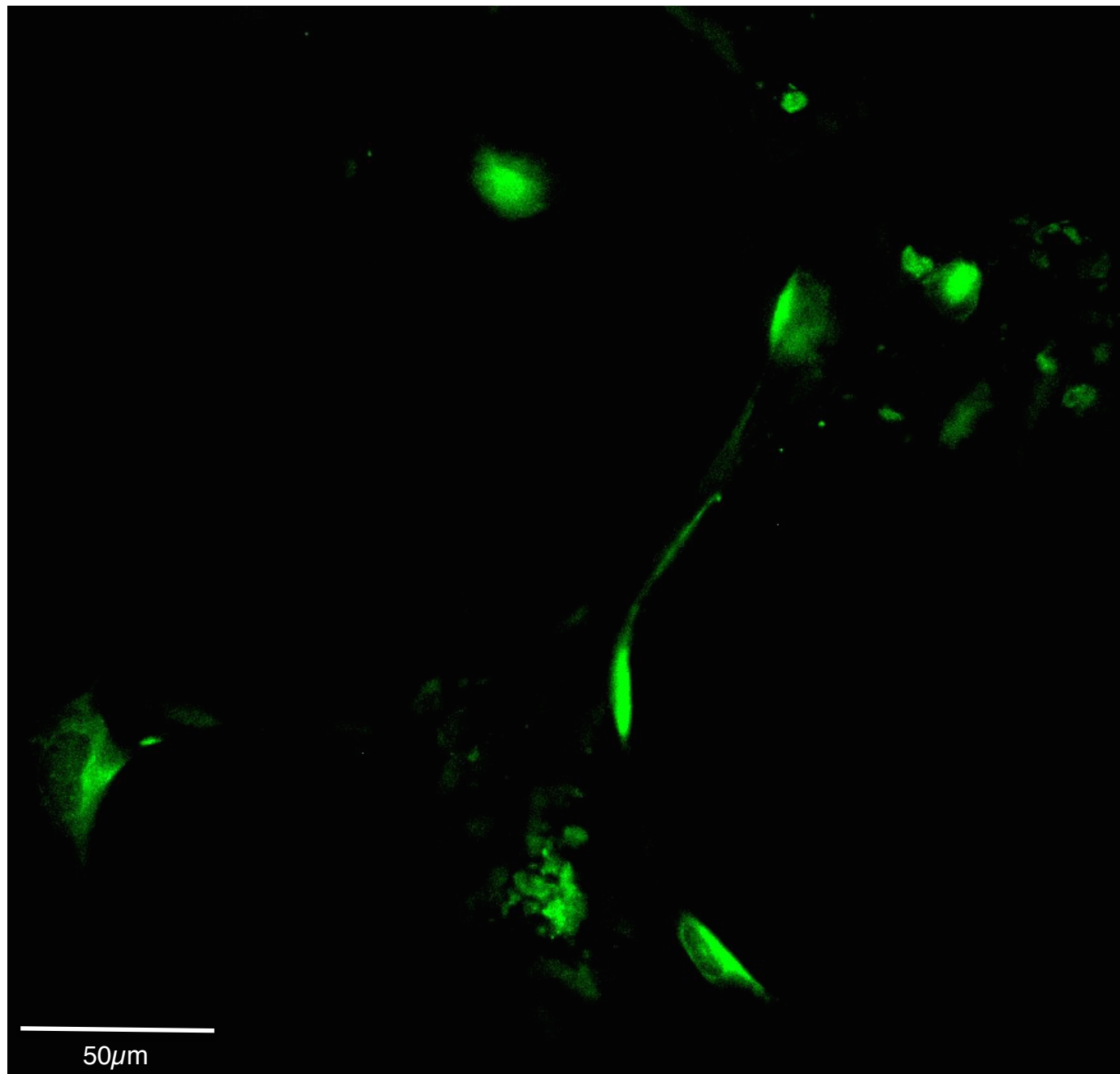

MyHC2

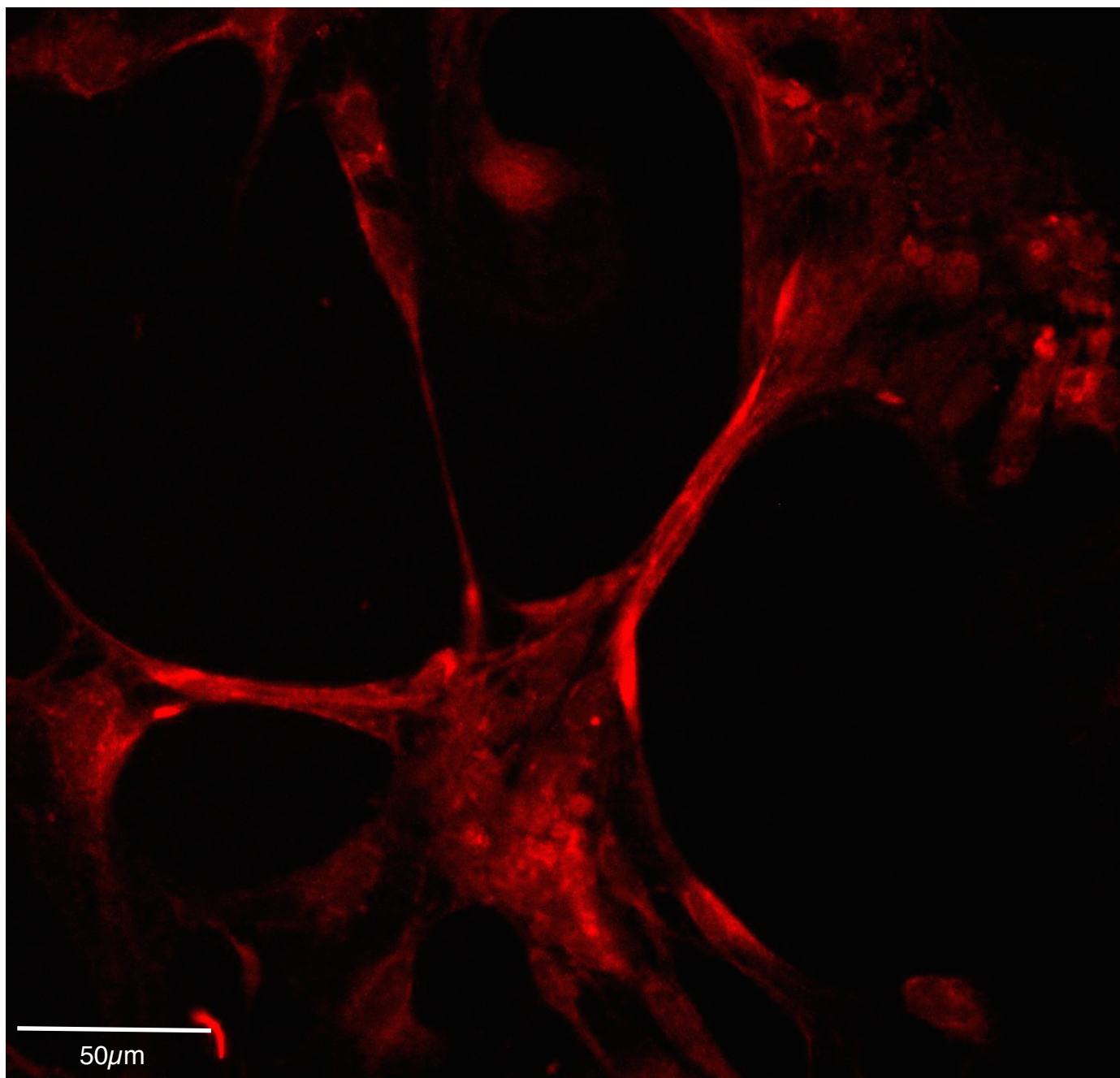

Supplement: Additional file 3: — Fluorescence microscopy of MyHC2 after 14d. Single stainings of MyHC2 in MSC and Mb co-cultures under HGF stimulation for 14 d. (a) Nuclear staining with DAPI. (b) GFP-transduced MSC in green colour. (c) Staining for MyHC2 with Alexa fluor 594 as secondary antibody. Scale bars represent 50 μm. Magnification 200x. (PDF 1334 kb) [file 12860_2017_131_MOESM3_ESM.pdf]

DAPI

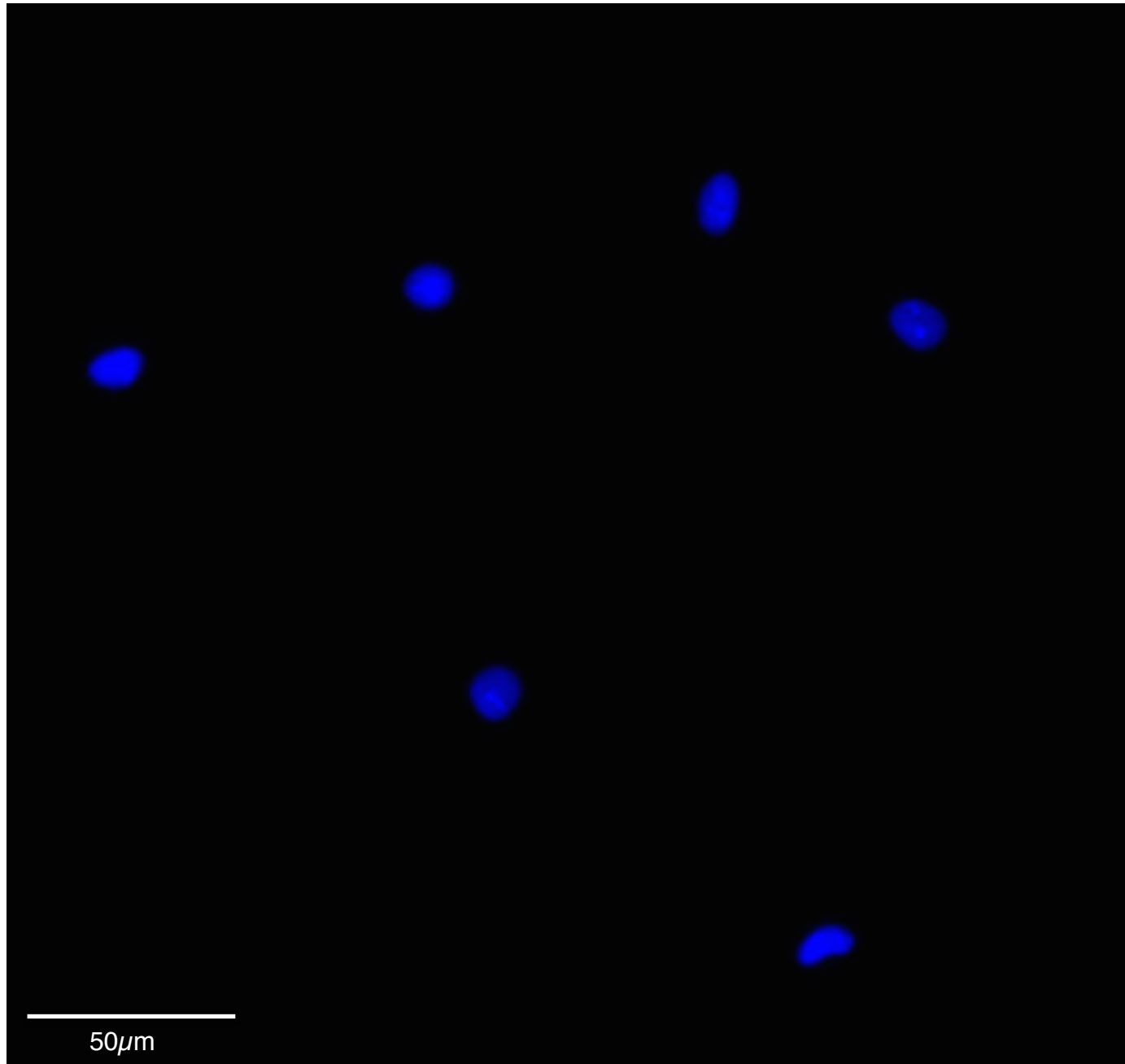

GFP

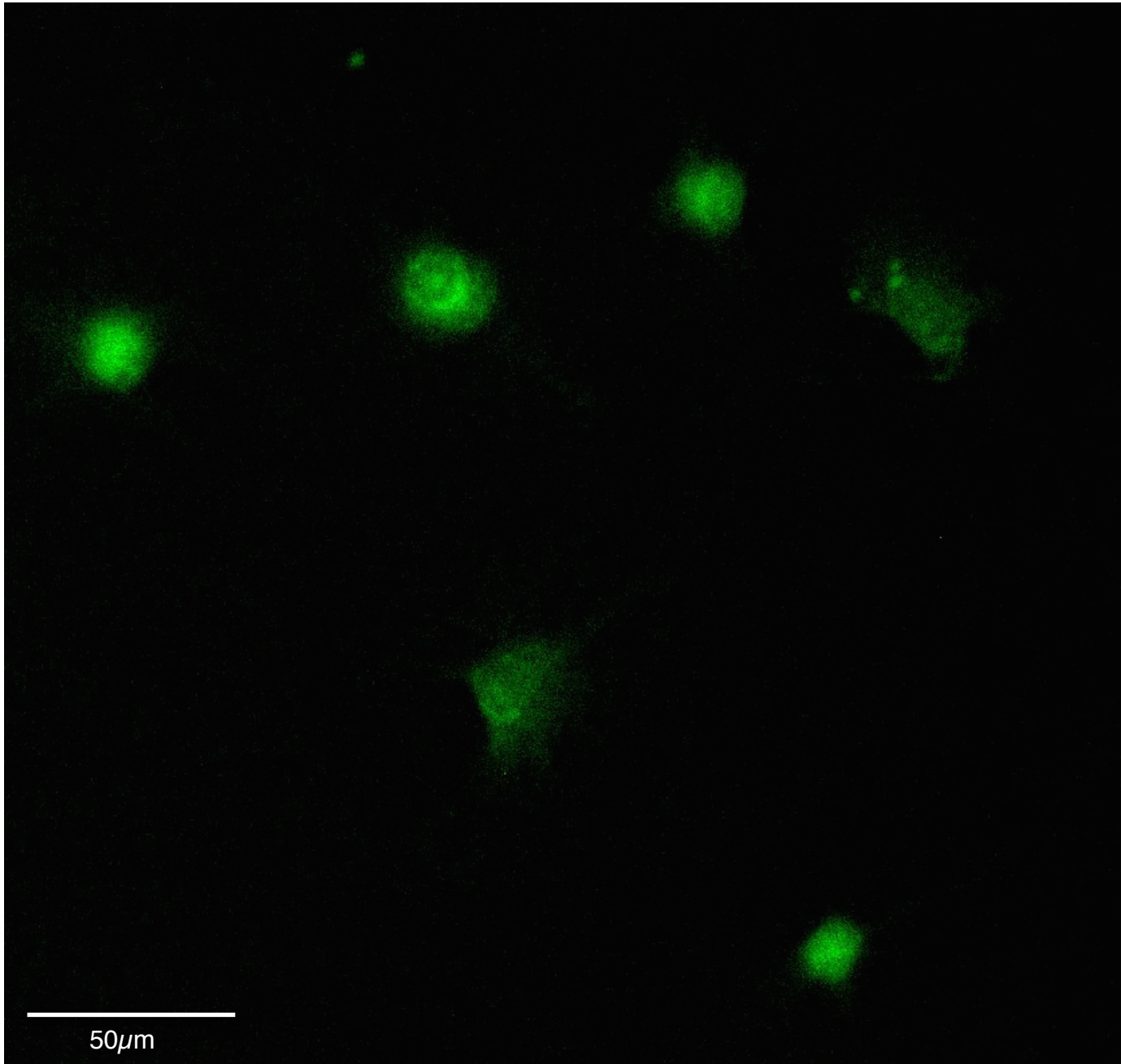

MEF2

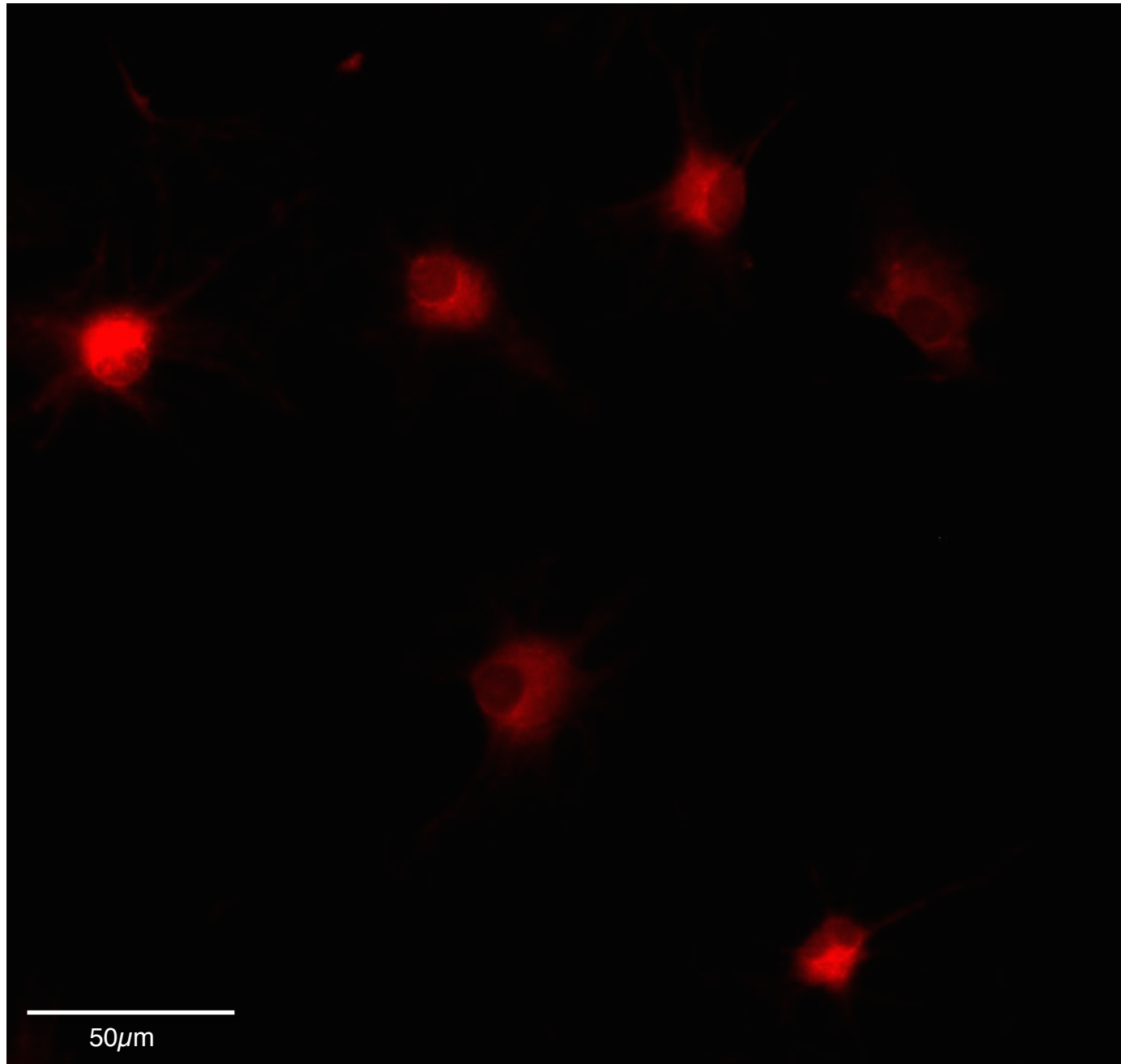

Supplement: Additional file 4: — Fluorescence microscopy of MEF2. Single stainings of MEF2 in MSC and Mb co-cultures in control groups without HGF/IGF-1 after 14 d. (a) Nuclear staining with DAPI. (b) GFP-transduced MSC are in green colour. (c) Staining for MEF2 with Alexa fluor 594 as secondary antibody. Scale bars represent 50 μm. Magnification 200x. (PDF 792 kb) [file 12860_2017_131_MOESM4_ESM.pdf]
